# Association between maternal postpartum depressive symptoms, socioeconomic factors, and birth outcomes with infant growth in South Africa

**DOI:** 10.1038/s41598-023-32653-x

**Published:** 2023-04-07

**Authors:** Hannah Ricci, Regina Nakiranda, Linda Malan, Herculina S. Kruger, Marina Visser, Cristian Ricci, Mieke Faber, Cornelius M. Smuts

**Affiliations:** 1 North-West University (Centre of Excellence for Nutrition), Potchefstroom, South Africa; 2 North-West University (Africa Unit for Transdisciplinary Health Research (AUTHeR)), Potchefstroom, South Africa; 3 South African Medical Research Council (Non-Communicable Diseases Research Unit), Tygerberg, South Africa

**Keywords:** Psychology, Risk factors

## Abstract

This study aimed to investigate the association between maternal postpartum depressive symptoms, household demographic, socioeconomic, and infant characteristics with infant physical growth, and how these factors correlate to determine latent factors. This study was based on the baseline data of a 6-month randomised controlled trial aimed at providing an egg a day to infants aged 6 to 9-months from a low socioeconomic community in South Africa. Information collected on household demographic, socioeconomic, and infant characteristics was by face-to-face structured interviews, and trained assessors took anthropometric measurements. The Edinburgh Postnatal Depression Scale (EPDS) was used to assess maternal postpartum depressive symptoms. The analysis was based on 428 mother-infant pairs. Total EPDS score and its subscales score were not associated with stunting or underweight risk. However, a three- to four-fold increased risk of stunting and underweight, respectively was observed for premature birth. Low birthweight was associated with an estimated six-fold increased risk of underweight and stunting. Being female was associated with about 50% reduced risk of stunting and underweight. In conclusion, more robust studies are needed to substantiate these findings, with more awareness creation on the consequences of LBW and prematurity on the physical growth of infants from resource-limited settings.

## Introduction

Maternal postpartum depression is estimated to affect 10–19% of women in high-income and low- and middle-income countries (LMICs), respectively^1^. It is a non-psychotic sub-type of major depressive disorder, which usually occurs within 1–12 months of childbearing^2,3^. Symptomatic features include sadness or irritable mood, loss of interest or pleasure in all or almost all activities, decrease or increase in appetite, difficulty sleeping or oversleeping, and feeling of restlessness or being slowed down; other symptoms include tiredness or loss of energy, feelings of worthlessness or guilt, decreased ability to think or concentrate or indecisiveness, as well as psychomotor changes, such as agitation or retardation and suicidal thoughts^4^. If left untreated, it can have negative and severe long-term consequences on the mother, her children, and her relationship with others^3,5^. In LMICs, the presence of maternal postpartum depressive symptoms has shown to be associated with childhood undernutrition such as stunting and underweight^6,7^. This is because the presence of maternal postpartum depressive symptoms may interfere with a mother's ability to adequately care for her infants and young children^7^. There have been reports that mothers who show signs of depression during the postpartum period are less likely to initiate and continue breastfeeding, and they are less likely to provide an adequate diet for their children. This may expose these vulnerable infants and young children to suboptimal growth as they mostly depend solely on their mothers for their nutritional and social needs from birth^5,8^.

Studies that evaluated the association between maternal postpartum depression and infant physical growth outcomes in LMICs have produced inconsistent results. For instance, several studies have shown that infants and young children of mothers with postpartum depression are at increased risk of stunting and/or underweight^5,6,9–17^. In contrast, other studies showed no association between maternal postpartum depression and infant growth including stunting, weight and height or length^18,19^. An explanation for the difference in results could be the study design, age group of the study participants, screening tools, diagnostic criteria, residual confounding given by the lack or minimal adjustment for confounders, and the language used during screening^5,6^. Another plausible explanation for the difference in findings may have been socioeconomic and sociocultural factors, such as poverty, food insecurity, maternal education and marital status, which may determine the association between maternal depression and infant nutritional status^20^. Nevertheless, previous^21,22^ and recent^23^ meta-analyses, the strongest form of scientific evidence, provided a summary of research findings and demonstrated that an association exists between maternal postpartum depression and infant physical growth.

Despite the clinical, social, and public health significance of maternal postpartum depressive symptoms, there is a paucity of evidence of other dimensional mechanisms linking maternal postpartum depressive symptoms with poor infant physical growth. Firstly, there is no study that seeks to investigate how the instruments used for screening maternal postpartum depression associate with infant physical growth outcomes. For example, the Edinburgh Postnatal Depression Scale (EPDS), which includes a 10-item self-reporting scale has been generally employed as a screening tool during the postpartum period; this tool has been validated for use in LMICs and in South Africa^24,25^. However, there are limited studies on how the EPDS scores, and its subscales associate with poor infant growth, including stunting and underweight. There is also lack of evidence on how household socioeconomic status and infant characteristics associate with maternal postpartum depressive symptoms and poor infant physical growth. Furthermore, no studies have evaluated how the individual 10-items of the EPDS associate with infants’ physical growth. Therefore, the main purpose of this study was to investigate whether maternal postpartum depressive symptoms associate with infant physical growth in a low socioeconomic community in South Africa. Secondly, this study investigated how maternal postpartum depressive symptoms, household demographic, socioeconomic, and infant characteristics correlate to determine latent factors, and how the latent factors associate with infants’ physical growth in a low socioeconomic community in South Africa. Finally, this study estimated the direct association of all characteristics under investigation with the risk of stunting and underweight.

## Materials and methods

### Study design, setting and recruitment of participants

The basis of this study was the baseline assessment of a 6-month randomised controlled trial (RCT) of an egg daily as a complementary food to infants aged 6 to 9-months, living in the peri-urban Jouberton Klerksdorp, City of Matlosana, Dr Kenneth Kaunda District Municipality, North West Province, South Africa. With an area of 19 km^2^, Jouberton has a population of 114,256 with a median age of 25.2 years, a human development index of 0.6109^26^, and with the most spoken language being Tswana. The baseline data was collected from 16 February 2021 to 7 July 2021.

Mother-infant pairs were recruited mainly at household level using information sheets, posters and pamphlets. Mothers who were willing to take part in the study were invited to the central study site for consent, screening, and enrolment. Infants were eligible to take part in the study if they were 6 to < 9-months of age, and if they resided in the study area. Infants were excluded if they had severe obvious congenital abnormalities, haemoglobin (Hb) < 7 g/dL, a weight-for-length Z-score less than − 3, self-reported diseases referred for hospitalisation by a health professional, known allergies/intolerances to eggs, were receiving special nutritional supplements as part of a feeding programme, and if not born as a singleton. Infants were also excluded if the mother had plans to move out of the study area in the next 9 months and if the mother was below 18 years old at the start of the study.

### Data collection procedures

Information collected on household characteristics, infant feeding practices and maternal postpartum depressive symptoms was by a face-to-face structured interview by trained assessors, in a quiet room at the central study site, in the local language of the mother. All anthropometric measurements took place at the central study site. Data collection was by the trained assessors throughout the study. A professional nurse collected a capillary blood sample by heel prick for Hb analysis to determine the prevalence of anaemia based on Hb < 11 g/dL. The Hb was adjusted for altitude using an adjustment factor of − 0.2 g/dL^27^.

### Household demographic and socioeconomic characteristics

Demographic information included maternal age, educational status, marital status, and the number of people in the household. Information on household socioeconomic status, such as the number of people employed and earning a salary, number of people receiving a social grant, if they went hungry in the past seven days due to shortage of money, source of drinking water, type of toilet and the availability of electricity were also collected.

### Infant feeding practices

Information on current breastfeeding status, age of introduction of complementary foods, such as liquid, semi-solids, and solids, as well as whether the infant was receiving any dietary supplements, was collected based on the World Health Organization’s (WHO) guidelines for assessing Infant and Young Child Feeding practices^28,29^.

### Maternal postpartum depressive symptoms assessment

Maternal postpartum depressive symptoms were assessed using the 10-item Edinburgh Postnatal Depression Scale (EPDS), as it has been previously validated in a cohort of South African women^25^. The EPDS assesses the following symptomatic characteristics of a postpartum major depressive episode, namely, loss of interest or pleasure, self-blame, feeling anxious, feeling sad, tearfulness/crying when unhappy, difficulty sleeping, and thoughts of death^4^. The mothers rated the frequency of symptoms that corresponds to their feelings in the last seven days before the survey. The frequency of symptoms was graded 0 if it “never” occurred, 1 if it occurred “occasionally” or “hardly” or “not very often”, 2 if it occurred sometimes, and 3 if it occurred “most of the time” or “quite often or very often”. The total score on the EPDS may range from 0 to 30. A score of ≥ 10 and ≥ 13 was recommended in the original validation study to indicate possible depression and probable major depressive illness, respectively. A total score higher than 13 may indicate that more depressive symptoms are being expressed^30^. In view of these, a cut-off of ≥ 10 and ≥ 13 was used in this study. Three more subscale scores were further considered^31^. A first subscale (anxiety scale) was computed by the sum of 3 EPDS items regarding self-blame, being anxious and/or worried and being scared and/or panicky. A second subscale (depression scale) was computed by the sum of the other 7 EPDS items. The computing of a third subscale was by the sum of two EPDS items that resemble the Patient Health Questionnaire-2 (PHQ-2) regarding enjoyment and feeling sad or miserable, respectively. The original PHQ-2 is a two-question questionnaire which asks the following questions on a scale of 0 to 3: I’ve had little interest or pleasure in doing things, and I’ve been feeling down, depressed, or hopeless. The subscales were rescaled to the same numerical metric of the overall EPDS by multiplying the subscale score by a constant given by 10, divided by the number of items that made up that score^31^.

### Anthropometric assessments

The infant’s date of birth, birthweight, and birth length, as well as maternal gestational age were recorded from the infant’s clinic booklet; however, in the absence of such information in the booklet, maternal recall of gestational age was used. Low birthweight (LBW) was defined as birthweight < 2.5 kg and prematurity as gestational age < 37 weeks. Fieldworkers trained according to the WHO’s training course on child growth assessment^32^ measured the infants’ weight and length. Infants were undressed and weighed to the nearest 0.001 kg using two standardised digital infant scales (Seca 334 and 727). Recumbent length was measured to the nearest 0.1 cm using an infantometer (Seca 416). If the first two measurements taken differed by more than 0.01 kg for weight and 0.5 cm for length, a third and/or a fourth measurement was done, and the average of the two closest values taken. Infants’ anthropometric measurements were converted to length-for-age (LAZ) and weight-for-age (WAZ) Z-scores using the WHO’s child growth standards^33^ of the SAS software. Stunting was defined as LAZ < − 2 and underweight as WAZ < − 2. Mothers’ height and weight were measured to calculate their body mass index (BMI).

### Statistical methods

Participants’ characteristics were described using median and interquartile range (IQR) or count and percentage (%). Univariate comparisons were performed using the non-parametric Mann–Whitney U-test or the Chi-square test for continuous and categorical variables respectively. The Fisher’s exact test was applied instead of the Chi-square when one or more of the cell counts was below five. A latent factor model was performed to investigate the complex relationship linking household demographic, socioeconomic, and infant characteristics to infant physical growth through maternal postpartum depressive symptoms. Our model had the EPDS total score as a mediator of the relationship between household demographic, socioeconomic, and infant characteristics. Supplementary Fig. 1 portrays the graphical model considered. Additional analysis was performed based on EPDS subscales in relation to the prevalence of stunting and underweight. Finally, a binomial-based generalised linear model, with logarithmic link adjusted for mother’s age, was adopted to estimate the risk of stunting and underweight given by the direct action of the EPDS overall score and by anxiety and depression subscale medians.

All statistical tests were two tailed. Type-I error to detect significant associations was defined according to the Benjamini–Hochberg procedure^34^ to minimise the false discovery rate due to numerous tests without compromising the false negative rate (type-II error). Statistical evaluations were performed using the R statistical software version 4.1.2. The Lavaan package was used to perform simultaneous regressions to interpolate the latent factor model; generalized linear models (GLMs) were performed by using the GLM function.

#### Assessment of the sample size adequacy

Two power calculations were performed considering a range of possible type-I error rates defined according to the Benjamini–Hochberg procedure. The first power post-hoc calculation was for the latent factor model, investigating household demographic and socioeconomic characteristics in relation to infants’ physical growth through postpartum depressive symptom and breastfeeding status. Based on a Root Mean Standard Error (RMSE) of 0.05, a type I error rate in the range of 0.1–0.05% (0.0005 < α < 0.001), a sample size of 313 to 330 participants is sufficient to guarantee a type-II error rate below 10% (1 − β > 0.8) in a structural equation model with more than 100 degrees of freedom. The second post-hoc power calculation performed was for the binomial function, estimating the risk of stunting and underweight. Given a type I error rate in the range of 0.1–0.05% and a risk ratio (RR) estimate greater than 1.5, a sample size of 350–400 participants would confirm a type-II error or a false negative rate below 10% (statistical power 1 − β ≥ 0.9). The semPower and the pwr packages of the R software were used for the power calculation of the latent factor model and binomial regression, respectively.

### Ethics approval

This paper forms part of a PhD thesis. The performing of the study was in line with the principles of the Declaration of Helsinki. The Health Research Ethics Committee of the North-West University (NWU-00452–19-A1) granted approval. The RCT was registered on clinicaltrials.gov (NCT05168085).

### Consent to participate

Written informed consent was obtained from all mothers/caregivers included in the study.

## Results

### Household demographic and socioeconomic factors

The overall study was based on 500 mother/caregiver-infant pairs. In the present study, 57 records were excluded because the respondents were not the biological mothers of the infants. Additional two records were excluded due to mothers’ pregnancy status, which could have biased the retrospective assessment of postpartum depressive symptoms. Finally, 13 more records were excluded due to missing values of variables under investigation. The analytical sample thus resulted in 428 infants, 206 boys and 222 girls. Table 1 presents the household demographic, socioeconomic and EPDS characteristics of the infants stratified by stunting and underweight. In the total group, the median (IQR) age of the infants was 198 (187, 230) days, length was 65 (63.1, 66.8) cm, weight was 7.7 (6.8, 8.3) kg; 25.5% of infants were stunted, and 10.5% were underweight. The median (IQR) birthweight was 3.1 (2.7, 3.3) kg and gestational age was 39 (38, 40) weeks, both of which were significantly lower in both stunted and underweight infants (P < 0.0001). Overall, the median (IQR) Hb was 11.7 (11.0, 12.4) g/dL and 30.6% (n = 131) of infants had Hb below 11.0 g/dL. In the total group, 29.0% (n = 124) of the infants were never breastfed and 7.7% (n = 33) had received dietary supplement at the time of the study.

**Table 1:** Characteristics of infants, mothers, and households

	All participants (n = 428)	No stunting (n = 319)	Stunting (n = 109)^b^	No underweight (n = 383)	Underweight (n = 45)^c^
Infants					
Age (days)	198 (187, 230)	197 (186, 230)	203 (187, 228)	198 (186, 229)	201 (187, 233)
Girls n (%)	222 (51.9)	176 (55.2)	46 (42.2)*	204 (53.3)	18 (40.0)°
Birthweight (kg)	3.1 (2.7, 3.3)	3.2 (2.8, 3.4)	2.8 (2.3, 3.2)***	3.1 (2.8, 3.4)	2.5 (2.1, 3.0)***
Gestational age (weeks)	39.0 (38.0, 40.0)	40 (38, 40)	38 (36, 40)***	39.3 (38, 40)	37 (35, 39)***
Haemoglobin (g/dL)	11.7 (11.0, 12.4)	11.6 (10.8, 12.2)	11.3 (10.4, 12.0)	11.5 (10.8, 12.2)	11.4 (10.4, 11.8)
Anaemia (haemoglobin < 11 g/dL) n (%)	131 (30.6)	90 (28.2)	41 (37.6)°	114 (29.8)	17 (37.8)
Never breastfed n (%)	124 (29.0)	95 (29.8)	29 (26.6)	112 (29.2)	12 (26.7)
Any dietary supplement use n (%)	33 (7.7)	23 (7.2)	10 (9.2)	27 (7.1)	6 (13.3)
Mothers					
Age (years)	27.0 (23.0, 33.0)	27.0 (22.0, 33.0)	28.0 (23.0, 33.0)	27.0 (23.0, 33.0)	28.0 (22.0, 34.0)
Body mass index (kg/m^2^)	27.3 (22.3, 32.8)	27.6 (22.9, 33.2)	26.7 (22.1, 31.8)	27.6 (22.8, 33.2)	25.7 (20.2, 29.5)
Education below grade 10	140 (32.7)	96 (30.1)	44 (40.4%)*	119 (31.1)	21 (46.7)°
Not married, separated or widowed	255 (59.6)	192 (60.2)	63 (57.8)	232 (60.6)	23 (51.1)
Household sociodemographic and socioeconomic factors					
> 5 people in the household	181 (42.3)	126 (39.5)	55 (50.5)*	161 (42.0)	20 (44.4)
> 1 primary school child	118 (27.6)	77 (24.1)	41 (37.6)**	102 (26.6)	16 (35.6)
> 1 child below five years	37 (8.6)	26 (8.2)	11 (10.1)	35 (9.1)	2 (4.4)
> 1 elderly person	80 (18.7)	61 (19.1)	19 (17.4)	71 (18.5)	9 (20.0)
At least one person is employed	249 (58.2)	191 (59.9)	58 (53.2)	225 (58.8)	24 (53.3)
At least one person earns a social grant	310 (72.4)	230 (72.1)	80 (73.4)	279 (72.9)	31 (68.9)
Absence of own tap water in/outside	32 (7.5)	25 (7.8)	7 (6.4)	30 (7.8)	2 (4.4)
Absence of electricity	58 (13.6)	44 (13.8)	14 (12.8)	52 (13.6)	6 (13.3)
Absence of flush toilet	43 (10.0)	35 (11.0)	8 (7.3)	42 (11.0)	1 (2.2)°
Hungry due to shortage of money^a^	214 (50.0)	160 (50.2)	54 (49.5)	184 (48.0)	30 (66.7)*
Edinburgh Postnatal Depression Scale (EPDS)					
PDS: ANX score (continuous)	7 (0, 10)	7 (0, 10)	7 (0, 10)	7 (0, 10)	7 (0, 10)
EPDS: ANX score ≥ 10	140 (32.7)	108 (33.9)	32 (29.4)	126 (32.9)	14 (31.1)
EPDS: DEP score (continuous)	7 (4, 10)	7 (4, 10)	7 (3, 10)	7 (4, 10)	7 (4, 10)
EPDS: DEP score ≥ 10	131 (30.6)	97 (30.4)	34 (31.2)	118 (30.8)	13 (28.9)
EPDS: PHQ score (continuous)	15 (5, 20)	15 (5, 15)	15 (5, 20)	15 (5, 20)	15 (10, 15)
EPDS: PHQ score ≥ 10	317 (74.1)	236 (74.0)	81 (74.3)	280 (73.1)	37 (82.2)
EPDS: Total score (continuous)	6 (3, 10)	6 (3, 10)	6 (3, 10)	6 (3, 10)	7 (4, 10)
EPDS: Total score ≥ 10	122 (28.5)	91 (28.5)	31 (28.4)	110 (28.7)	12 (26.7)
EPDS: Total score ≥ 13	65 (15.2)	50 (15.7)	15 (13.8)	59 (15.4)	6 (13.3)

Chi-square and independent sample t-test were performed using categorical and continuous variables between stunting and underweight infants versus their healthy counterparts, ^a^in the past week, ^b^stunting defined as length-for-age Z-score < − 2; ^c^underweight defined as weight-for-age Z-score < − 2. Comparison of the rates of positive answers to EPDS to stunting and underweight children vs. their healthy counterparts ***p < 0.001, **p < 0.01, *p < 0.05, °p < 0.10.

Mothers had a median (IQR) age of 27 (23.0, 33.0) years with a median (IQR) BMI of 27.3 (22.3, 32.8) kg/m^2^. About a third of the mothers had education below grade 10 (32.7%; n = 140), and 59.6% (n = 255) were not married or were separated or widowed. Overall, 7.5% (n = 32) reported not having their own tap water either inside or outside the home, while 13.6% (n = 58) and 10.0% (n = 43) reported not having electricity and a flush toilet in the home, respectively. In the total group, 50.0% (n = 214) of the mothers reported being hungry in the past week due to a shortage of money. The proportion of mothers reporting to be hungry in the past week due to shortage of money was significantly higher for the underweight infants compared to their healthy counterparts (66.7% (n = 30) versus 48.0% (n = 184); P = 0.0181).

### Maternal postpartum depressive symptoms outcome

Overall, 28.5% (n = 122) of the mothers had a total EPDS score ≥ 10 and 15.2% (n = 65) had a total EPDS score ≥ 13. For the EPDS subscales, 32.7% (n = 140), 30.6% (n = 131) and 74.1% (n = 317) reported a score of ≥ 10 for anxiety, depression, and PHQ-2 subscales, respectively. When looking at the individual items of the EPDS, we found the number of mothers reporting negative feelings on the EPDS ranged from 6.5% (n = 28) for suicidal ideation to 86.7% (n = 371) for loss of enjoyment. More than half of the mothers reported not being able to laugh (50.9%; n = 218) and self-blame (52.8%; n = 226), almost half reported feeling anxious (43.0%; n = 184), not being able to cope (45.3%; 194) and feeling sad (49.3%; n = 211), while 26.9% (n = 115) reported feeling scared and 31.5% (n = 135) reported crying often. The internal consistency of the EPDS questionnaire in the sample was satisfactory, having a Cronbach alpha of 0.752.

### Analysis of the latent factor model for infants’ physical growth outcomes

**Figure 1:**
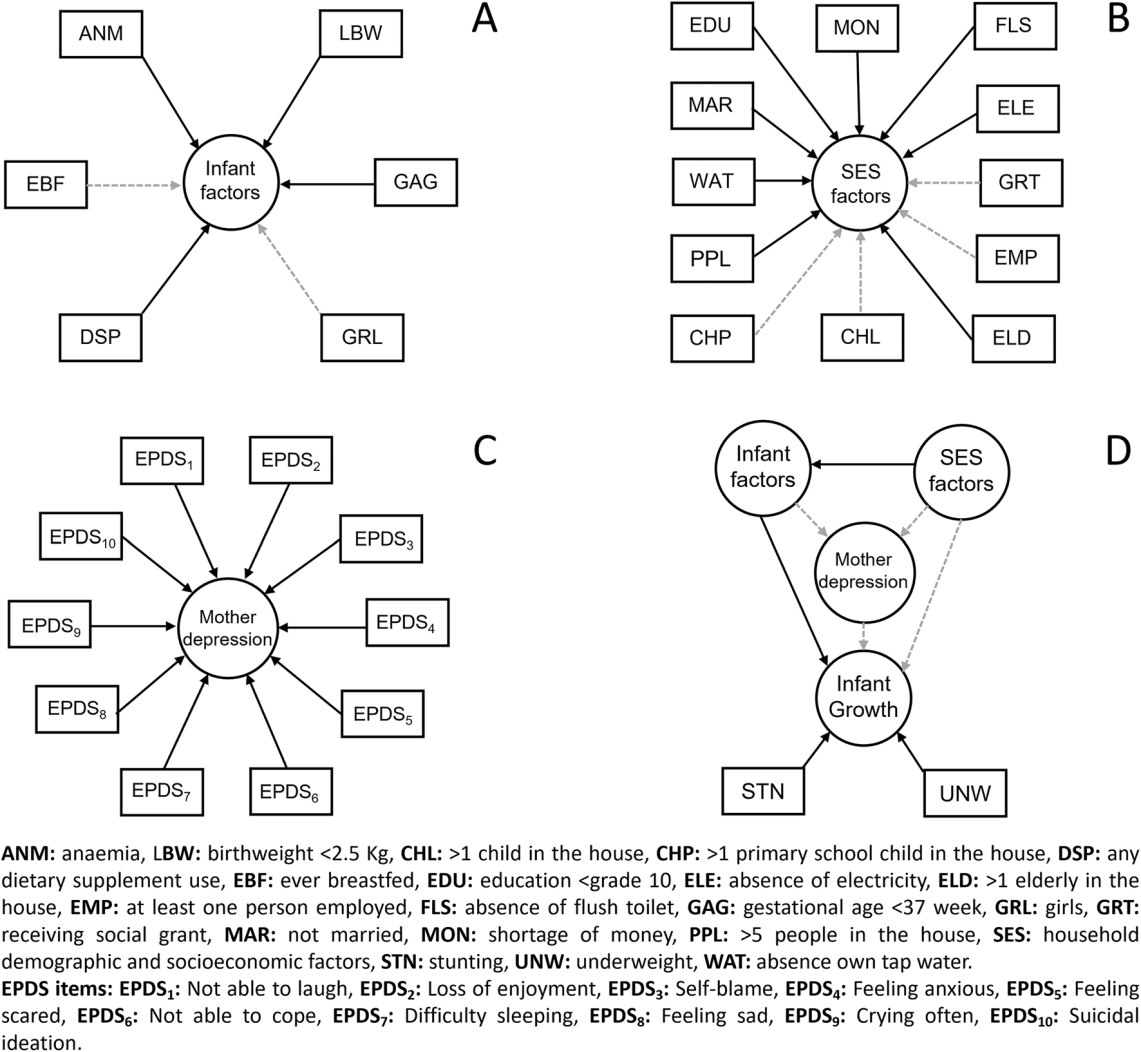
Results from the latent factor model analysis. Correlations between instrumental variables (Rectangles) and latent factors (circles) were reported on Panels (**A**), (**B**) and (**C**). Overall latent factor model was reported on panel (**D**). Solid black arrows represent statistically significant associations according to the Benjamini–Hochberg correction.

**Figure 2:**
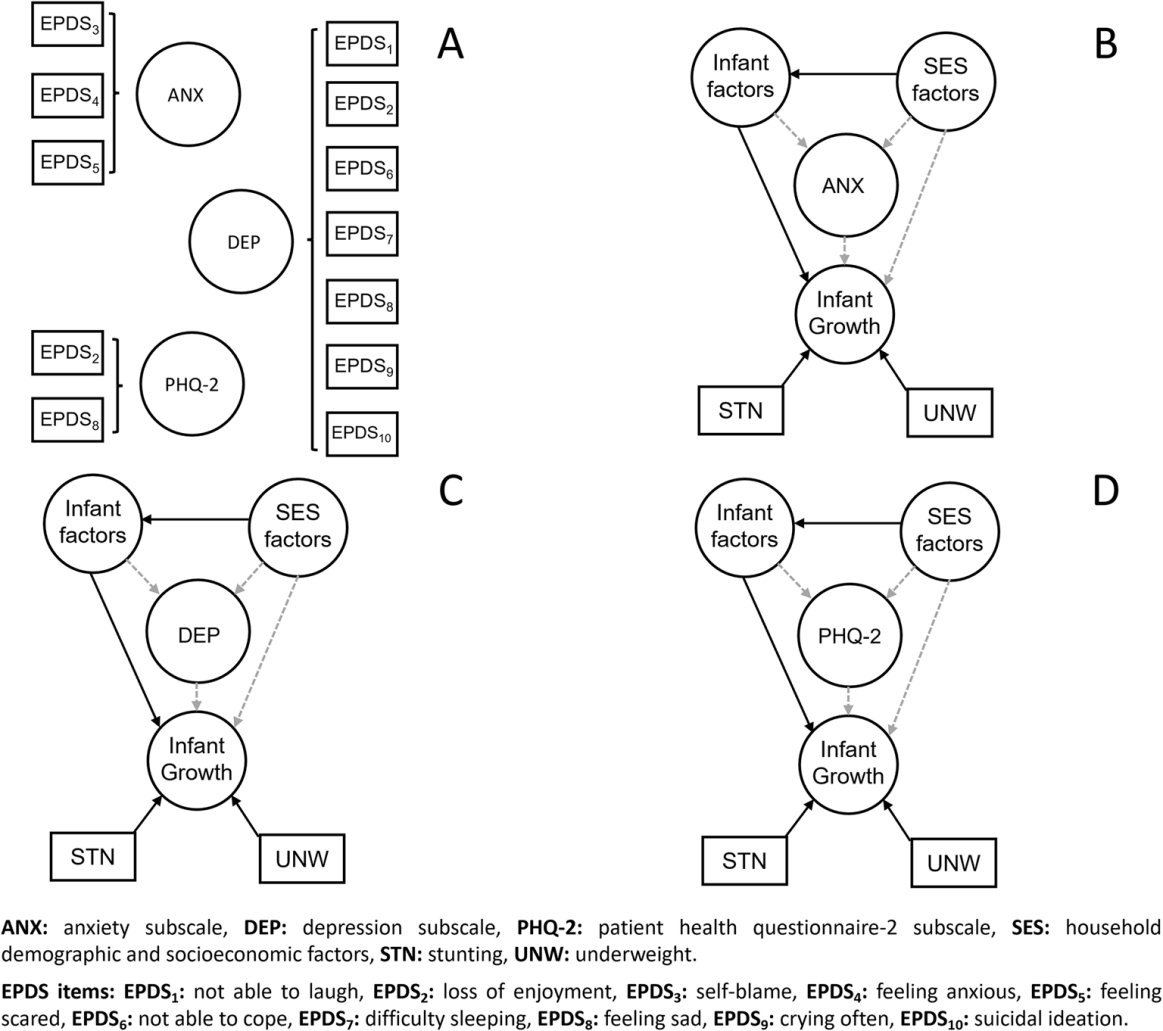
Latent factor model analysis applied to EPDS subscales. Definitions of subscales are reported on panel (**A**). Overall latent factor models are reported on panels (**B**), (**C**) and (**D**) for anxiety, depression, and patient health questionnaire-2 subscales, respectively. Solid black arrows represent statistically significant associations according to the Benjamini–Hochberg correction.

Figure 1 presents the latent factor model for maternal postpartum depressive symptoms, household demographic and socioeconomic factors, infant characteristics, and physical growth. When considering the correlation between variables and latent factors, we firstly observed a strong and statistically significant correlation for anaemia, LBW, prematurity, and the use of any dietary supplement (Fig. 1A). A statistically significant correlation was observed between all household demographic and socioeconomic factors, except for having more than one primary school child, having more than one child, receiving social grant, and having at least one person employed (Fig. 1B). Finally, a statistically significant correlation was observed among all 10 individual EPDS items (Fig. 1C). Given these three latent factors, there was a statistically significant correlation observed between the latent factor defined by household demographic and socioeconomic factors, and the latent factor defined by infant characteristics (Fig. 1D). The latent factor given by infant characteristics is statistically significantly correlated with infant physical growth. In contrast, the latent factor defined by household demographic and socioeconomic factors, and the latent factor defined by maternal postpartum depressive symptoms are not directly associated with infant physical growth. Likewise, the latent factor of household demographic and socioeconomic factors and the latent factor defined by infant characteristics do not associate with maternal postpartum depressive symptoms. These analyses were confirmed when considering the correlation between the individual EPDS subscales (Fig. 2A) with stunting and underweight as outcomes (Fig. 2B–D).

### Determinants of stunting and underweight risks

**Figure 3:**
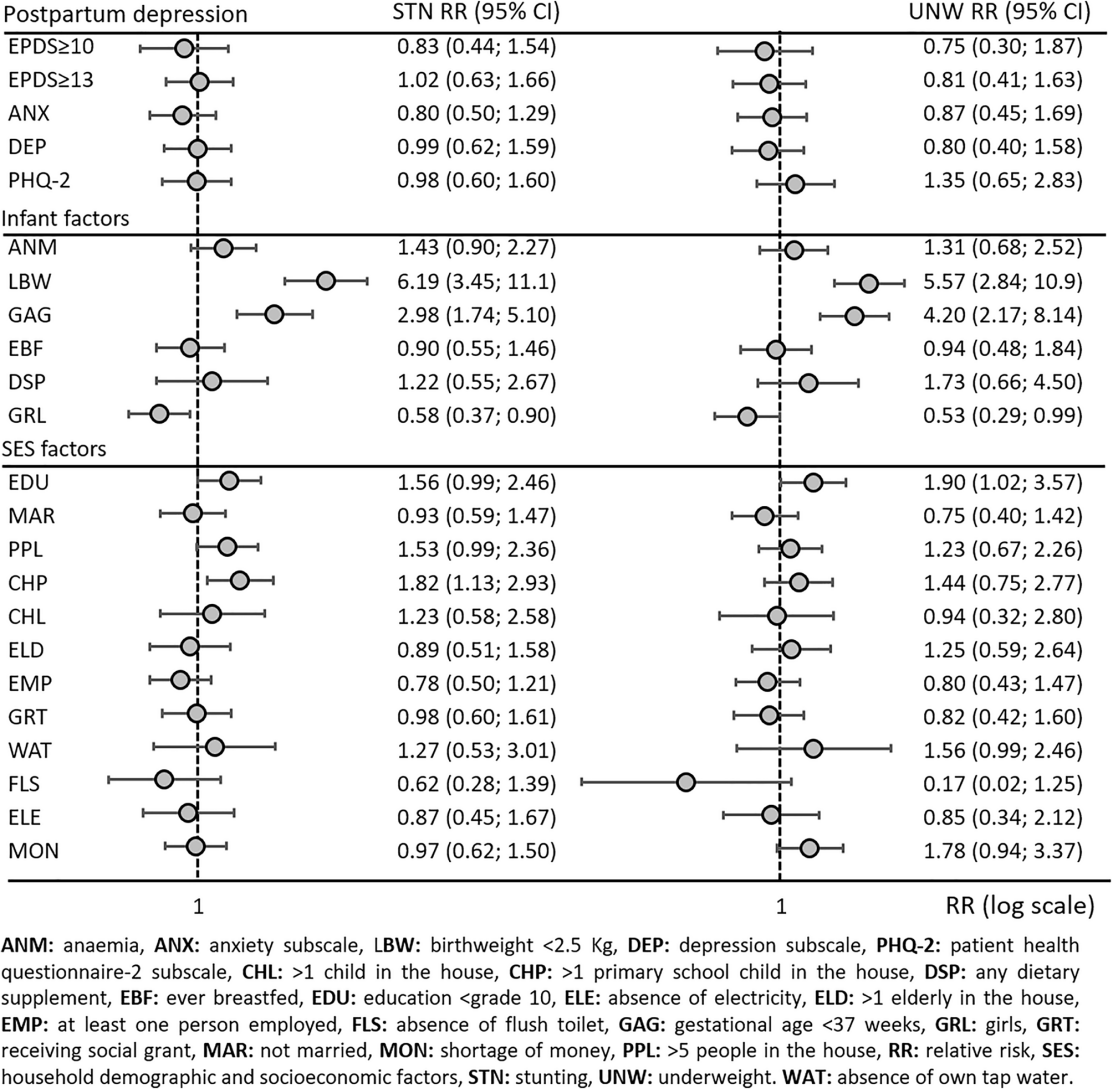
Association between all variables under investigation with stunting (STN) and underweight (UNW). Multivariate adjusted relative risk and 95% confidence intervals adjusted for mother’s age.

Figure 3 and Supplementary Fig. 2 present the risk factors for stunting and underweight. The overall EPDS score (≥ 10 or ≥ 13 as thresholds) and its subscales were not statistically significantly associated with stunting or underweight risk. Nevertheless, the EPDS item of crying often and feeling scared were statistically significantly associated with a twofold increased risk of stunting (RR 1.97; 95% CI 1.07, 3.63) and underweight (RR 2.03; 95% CI 1.00, 4.15), respectively. Regarding household demographic and socioeconomic factors, maternal education below grade 10 was associated with a borderline non-statistically significant 56% increased risk of stunting (RR 1.56; 95% CI 0.99, 2.46) and a statistically significant 90% increased risk of underweight (RR 1.90; 95% CI 1.02, 3.57). More than 80% increased risk of stunting (RR 1.82; 95% CI 1.13, 2.93) was observed for children in households with more than one primary school child. Furthermore, a borderline increased risk of stunting (RR 1.53; 95% CI 0.99, 2.36) and underweight (RR 1.78; 95% CI 0.94, 3.37) was observed for infants from households with more than five people and being hungry due to a shortage of money in the past week, respectively. Among infant characteristics, prematurity was associated with a threefold increased risk of stunting (RR 2.98; 95% CI 1.74, 5.10) and fourfold increased risk of underweight (RR 4.20; 95% CI 2.17, 8.14). LBW was associated with a sixfold increased risk of underweight (RR 5.57; 95% CI 2.84, 10.9) and stunting (RR 6.19; 95% CI 3.45, 11.1). There was also lower stunting (RR 0.58; 95% CI 0.37, 0.90) and underweight risk (RR 0.53; 95% CI 0.29, 0.99) was also observed for girls in comparison to boys. Although not statistically significant, a borderline increased risk of stunting (RR 1.43; 95% CI 0.90, 2.27) was observed for anaemia.

## Discussion

Generally, the total EPDS and its subscale scores did not appear to correlate with infant physical growth. Despite a lack of correlation between latent factors of maternal postpartum depressive symptoms, including its total and subscale scores with infant physical growth, we observed an association of certain individual EPDS items with stunting and underweight risk in a supplementary analysis. We observed a significant association between maternal depressive symptoms of crying often and feeling scared with stunting and underweight risk, respectively. Despite this observation, there has been no study conducted in this regard to directly assess this association. Conversely, some studies have shown that maternal anxiety and depression is associated with birth outcomes, such as LBW, prematurity, and poor child growth, such as stunting and underweight^13,35,36^. Reported, maternal postpartum depression is associated with increased risked of stunting in infants and young children, especially by the age of 2 years^10^. This support the findings of Surken et al.^7^ in its previous systematic review that infants and young children whose mothers are depressed or exhibit depressive symptoms are more likely to be stunted or underweight with a population attributable risk between 23 to 29%^7^. It is not yet well known the mechanisms for which maternal postpartum depressive symptoms associate with poor infant and young child growth. However, factors relating to feeding and care practices as well as the level food insecurity have been reported to play a role in this regard^7^. Coherently, three main characteristics of the general relationship between household demographic and socioeconomic, infant characteristics and maternal postpartum depressive symptoms in relation to infant physical growth emerged in our analysis. Firstly, it appears that a pattern of association exists between household demographic and socioeconomic factors, and infant characteristics such as anaemia, LBW, and prematurity. According to our latent factor model, household demographic and socioeconomic factors are not directly associated with maternal postpartum depressive symptoms and infant physical growth. Indirectly, it seems that household demographic and socioeconomic factors may act on infants’ growth at 6 to 9-months of age through a mechanism acting on LBW and prematurity^37^. In addition, we observed that infants from households with more than one primary school child had an increased risk of stunting. Although not statistically significant, the current study also showed that infants from households with many people and who went hungry in the past week due to a shortage of money were more likely to be stunted and underweight. This is in line with a study that reported that households with more family members were significantly associated with moderate to severe food insecurity^38^. It is also well documented that poverty usually coexists with food insecurity, poor nutrition, and poor access to water, hygiene, and sanitation^39,40^. Additionally, an association between poverty and undernutrition has been established in LMICs, including South Africa^41^. Therefore, the conclusion is that the consequences of poverty, such as food insecurity, and hunger are interrelated, and that they impact on nutritional status, thereby influencing growth and development of children^41,42^. Maternal education was also found to be a risk factor for infant stunting. This agrees with studies that report a strong relation between maternal education and child nutritional status, including stunting and underweight^43,44^. The reason for this finding may be that mothers who are educated may have a better knowledge about child health, identify childhood illnesses, seek treatment, read medical instruction, and are more receptive to modern medicine, as well as able to make informed choices about complementary feeding^45^.

Reportedly, infant characteristics, such as LBW and prematurity correlate with maternal postpartum depressive symptoms^46–48^. In contrast, we failed to find a direct correlation between LBW, prematurity, and maternal postpartum depressive symptoms. The speculation therefore is that the studies that found such an association may have used simple models with inadequate adjustment for confounders. This notwithstanding, it is widely acknowledged that having a preterm birth and/or LBW new-born is a stressful life experience^49,50^. Notably, having a LBW baby, and having to be hospitalised either due to the child being born with a LBW or born prematurely, can enhance the stress of the mother’s feeling about childcare and the inability to cope with life^48,51–53^. This may lead to the mother experiencing postpartum depressive symptoms, such as crying often and feeling scared, which this study found were also associated with stunting and underweight. It is thus recommended that mothers of LBW and premature infants be routinely screened and treated for postpartum depressive symptoms, and they should also be supported during these stressful life events^48,52^.

Again, the results of this study showed that infants who were anaemic had a borderline increased risk of stunting. Notably, infants and young children below the age of 2 years are at increased risk of developing anaemia due to their rapid growth and development during this period^54^; thus, iron deficiency anaemia has been reported to be very common in this age group, particularly in LMICs^54^. This may be because the usual complementary diets fed to these vulnerable children are often low in iron in terms of quantity and bioavailability, and the usual complementary foods given to infants normally contain inhibitors of iron absorption^54^. Anaemia has been reported to be associated with prematurity, LBW, and delayed child development^54^. Furthermore, the results of this study indicate that LBW and prematurity are risk factors for poor physical growth such as stunting and underweight in 6 to 9-month-old infants. This is congruent with other studies showing that LBW and prematurity are associated with increased risk of stunting and underweight in infants and young children^55–57^. In comparison to boys, the results of this study indicate that girls have a reduced risk of stunting and underweight. This is in line with previous^57–59^ and recent^60^ studies that reported that boys are at an increased risk of stunting and underweight. To date there is no established reason for this finding, as reportedly parents are willing to sacrifice more for their boy children than their girls during food insecurity and economic hardship, particularly in most LMICs^61,62^. Conversely, plausible explanations for our finding may be due to biological differences between boys and girls independent of feeding practices, susceptibility of boys to infections as well as the higher biological vulnerability of boys during infancy^59,63–65^. Thus, this could partially explain our finding as this study included only infants aged 6 to < 9-months.

One of the strengths of this study was that it first aimed to investigate the association between maternal postpartum depressive symptoms, household demographic and socioeconomic factors and infant physical growth in a peri-urban population in South Africa, a topic of relevant epidemiological and public health interest. Furthermore, this study was based on many participants, which means low false negative results and a high statistical precision of the estimates. The current study was based on data collected by using a validated tool such as the EPDS questionnaire. Another methodological strength of this study was that the same well-trained and experienced fieldworkers were responsible for data collection and capturing. Finally, this work adopted advanced statistical analyses, which represents the gold standard of tools, aimed at disentangling the complex relationship between numerous factors concurring to determine the outcomes under investigation. Additionally, this is the first study investigating the association between the individual 10-item EPDS symptoms and infant physical growth, such as stunting and underweight.

Coherently, this study was not free of limitations. Firstly, the cross-sectional nature of the study limited the assessment of causality, resulting in possible reverse causation. Nonetheless, such limitation appears as inconsequential because exposures were assessed backwards in time and because of the objective absence of any reverse causation. Conversely, we acknowledge that the retrospective assessment of the exposure might have generated recall bias; however, this was mitigated by adopting an approach based on structured interviews rather than self-reported records, although a structured interview does not necessarily eliminate recall bias. Secondly, despite the use of an advanced statistical approach, it could be assumed that the large number of statistical tests performed could have generated a problem of false discovery rate. This appears as a possible limitation, especially regarding the mediation analyses done. Notably, the Benjamini–Hochberg correction was applied to consider the false discovery rate. Alternatively, type-I error is not the only issue to be considered and this method was chosen over more rigorous alternatives because it effectively reduces the false discovery rate optimising the false negative rate as well. It is acknowledged that reducing the type-I error rate, and the false positives determining the so-called false discovery rate, inevitably increases the type-II error rate or risk of false negative results. In short, given the explorative, more than confirmatory nature of this study, the Benjamini–Hochberg procedure applied in this study appears as an optimal way to consider multiple testing and the risk of false negative rate at the same time. Another possible source of bias could be related to the COVID-19 pandemic as we cannot exclude that it might have biased the study. However, the data collection was performed during February to July 2021, during which the spread of COVID-19 was under control in our district and strict restrictions to human interaction were lifted. Also, all the national COVID-19 regulations and protocols were adhered to during data collection so that we may assume that the COVID-19 pandemic did not impact on the recruitment and baseline assessment process. All mitigation strategies were also in place as required by ethics. Furthermore, participants joined the study on a voluntary base possibly causing a selection bias. On the one hand, we assume this bias as negligible because of the large sample size of our study which, in turn, should improve the likelihood of the analytical sample to be representative of the target population. We acknowledge this as a potential limitation of any observational research based on volunteers. Finally, this study aimed at a very ambitious objective. Obviously, given the complex nature of the relation between maternal postpartum depressive symptoms, socioeconomic status and child physical growth, numerous pitfalls were unavoidable. Due to the novelty and explorative nature of this study, comprehensive studies with a fully prospective design, assessing maternal antenatal depressive symptoms before childbirth, are necessary to confirm the results of this study.

In conclusion, this study did not find any direct association between maternal postpartum depressive symptoms and poor infant physical growth, even when grouped into subscales. Despite this, a supplementary analysis of the 10 individual EPDS items showed that maternal depressive symptoms of crying often and feeling scared are associated with stunting and underweight risk, respectively. Additionally, the current study showed that infant characteristics, such as anaemia, LBW, prematurity, and boy sex are associated with stunting and underweight risk. This study showed that household demographic factors, such as family size, including number of primary school children, and maternal education, are risk factors for infant stunting and underweight. Thus, there is the need for more studies to establish the causal link between maternal postpartum depressive symptoms and poor infant growth to inform policy recommendations and practice. Additionally, there is the need for more global and context-specific strategies to address poor infant physical growth, such as stunting and underweight, particularly in LMICs including South Africa where undernutrition continues to be a public health concern.

## Supplementary Information

Supplementary Figure 1.

Supplementary Figure 2.

## Data Availability

The datasets generated and/or analysed during the current study are available from the corresponding author on reasonable request.
