# Supplementary figures and images for: Association between maternal postpartum depressive symptoms, socioeconomic factors, and birth outcomes with infant growth in South Africa
Source: Sci Rep. 2023 Apr 7;13:5696. doi: 10.1038/s41598-023-32653-x (PMC10080513; doi:10.1038/s41598-023-32653-x)

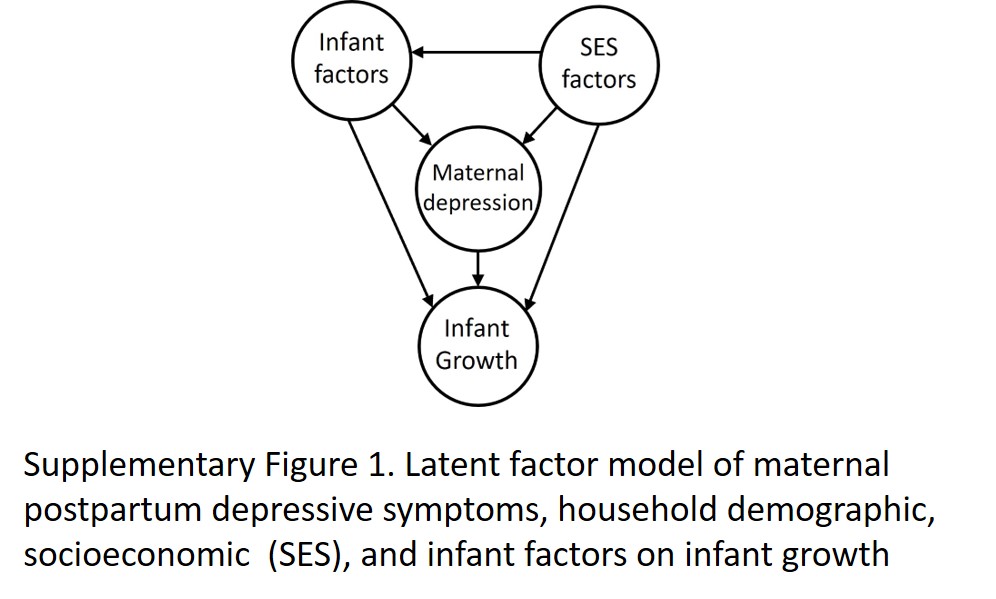

Supplement: Supplementary file 1 — Supplementary Figure 1. [file 41598_2023_32653_MOESM1_ESM.jpg]

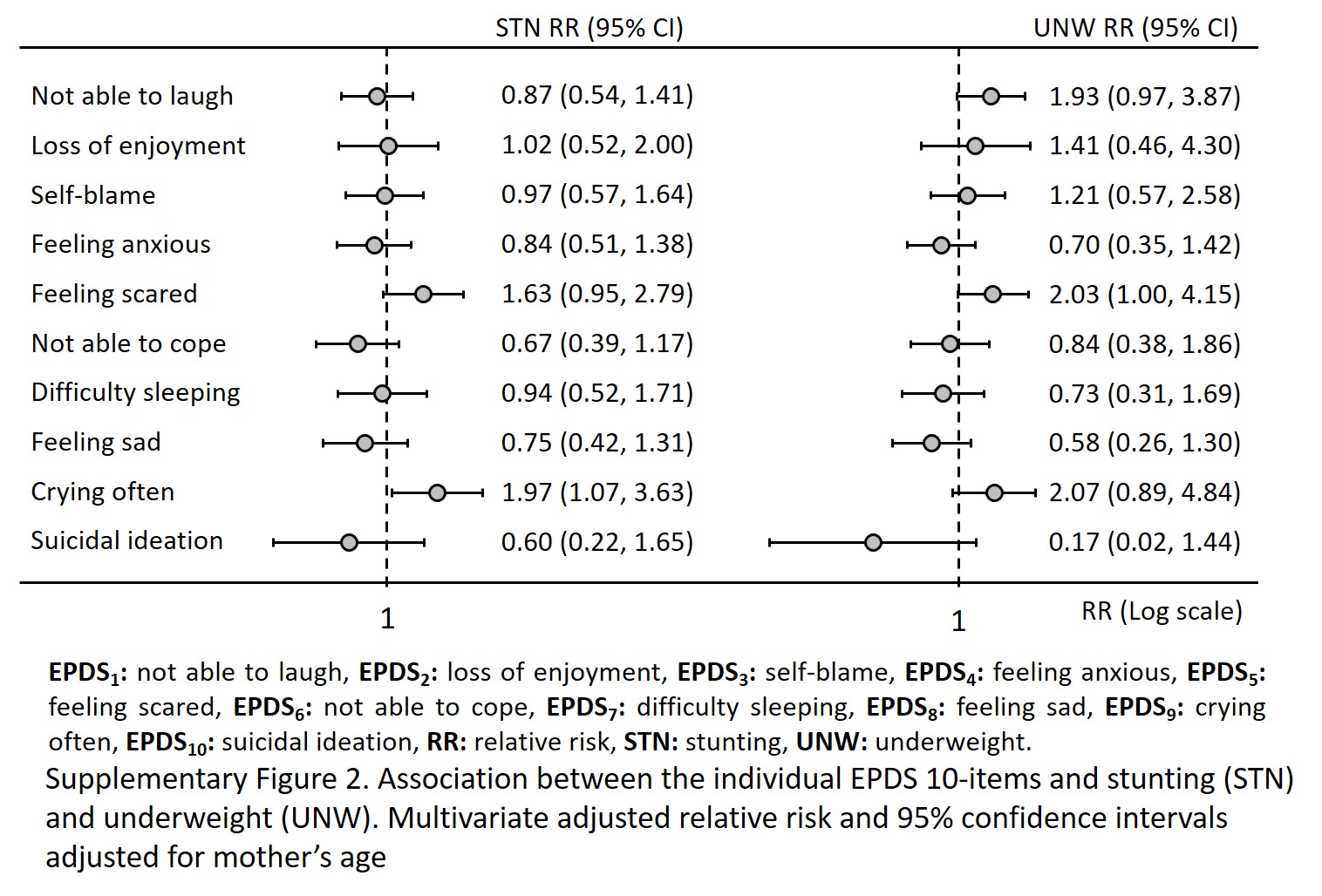

Supplement: Supplementary file 2 — Supplementary Figure 2. [file 41598_2023_32653_MOESM2_ESM.jpg]
